# Saturated Dissolved Oxygen Concentration in *in situ* Fragmentation Bioleaching of Copper Sulfide Ores

**DOI:** 10.3389/fmicb.2022.821635

**Published:** 2022-04-06

**Authors:** Ming-Qing Huang, Ming Zhang, Shu-Lin Zhan, Lin Chen, Zhen-Lin Xue

**Affiliations:** ^1^Zijin School of Geology and Mining, Fuzhou University, Fuzhou, China; ^2^College of Mining Engineering, North China University of Science and Technology, Tangshan, China

**Keywords:** *in situ* fragmentation bioleaching, copper sulfide ore, aeration, oxygen solubility, surface fitting

## Abstract

*In situ* fragmentation bioleaching is a promising way to perform deep mining safely, economically, and in an environmentally friendly manner, where oxygen plays a critical role in microbial growth and mineral dissolution. However, the lack of oxygen limits the implementation of in-situ fragmentation bioleaching. To overcome this limitation, aeration was proposed, with saturated dissolved oxygen concentration as an important indicator. Orthogonal experiments were conducted to measure saturated dissolved oxygen concentration at various temperature, pH, and electrolyte (ferrous sulfate, ferric sulfate, copper sulfate, and sulfuric acid) concentration conditions. Experimental data were analyzed by Python programming language and least squares method to obtain a saturated dissolved oxygen concentration model. Results showed that temperature had the most significant effect on oxygen solubility, which was concluded by comparing the results of surface fitting based on the least squares method. At 30–40°C, the saturated dissolved oxygen concentration decreased faster as metal ions concentration increased. The conjoint effect of the five variables on oxygen solubility showed that pH was linearly negatively related to oxygen solubility. Additionally, a mathematical model was also proposed to predict the saturated dissolved oxygen concentration in *in situ* fragmentation bioleaching of copper sulfide ores. This work enables bioleaching processes to be modeled and controlled more effectively.

## Introduction

*In situ* fluidized mining is an advanced technology used to achieve safe and efficient exploitation of deep and low-grade metal ore resources (Xie et al., 2019). *In situ* fragmentation bioleaching is a promising method used to realize fluidized mining of copper sulfide ores. It refers to *in situ* leaching of blasted ores in underground stopes with acidic solutions containing leaching microorganisms (Brierley, 2008; Brune and Bayer, 2012; Sinclair and Thompson, 2015; Peng et al., 2016; Johnson et al., 2021), and it is also related to indirect *in situ* leaching with biogenic lixiviants (Pakostova et al., 2018). During bioleaching, gas flow exerts great influence on metal recovery rate and microorganism activity. Gas flow provides oxidant O_2_ for the chemical reaction of minerals and leaching solutions, adjusts the temperature and oxygen concentration distribution of blasted heaps, and provides necessary O_2_ and carbon source for microbial growth (Dunbar, 2017). A dissolved oxygen concentration between 1.5 and 4.1 mg/L was required for optimal microbial activity (de Kock et al., 2004). Furthermore, the growth yield of *Acidithiobacillus ferrooxidans* on oxygen was 1.15 × 10^11^ cells/g (Ceskova et al., 2002). However, low oxygen concentration in deep environments is a widespread phenomenon that restricts the application of bioleaching. Due to the long heap leaching period and the drastic increase in aeration resistance in deep mines, oxygen is difficult to diffuse throughout the heaps by natural convection. The limitation of oxygen results in serious suppression of the growth of acidophilic and aerobic autotrophic microorganisms and bioleaching process of copper (Young, 1992; Li et al., 2021). For this reason, dissolved oxygen concentration becomes a limiting factor for ore bioleaching (Richter et al., 2017). Casas et al. (1998) found that oxygen diffuse distance to the heaps was within 5 m only by natural diffusion, and Mandl et al. (2014) studied the biochemical and biotechnological aspects of aeration conditions in iron- and sulfur-oxidizing *A. ferrooxidans* cultures. Thus, forced aeration is an important application to increase the leaching rate of ore by increasing oxygen supply (Chen et al., 2022), but there are still some problems such as low effective air volume rate, high power consumption, and high aeration cost. Therefore, effective aeration is a key point that needs to be considered when designing such a process, and the saturated dissolved oxygen concentration in bioleaching solutions at each bioleaching period is an important indicator.

*Acidithiobacillus ferrooxidans*, which is a strictly aerobic, obligate, and chemolithotrophic microorganism, has been used to leach bacteria in most bioleaching experiments (Tavakoli et al., 2021). The bacterium obtains energy for growth by the oxidation of inorganic substances such as ferrous iron, elemental sulfur, and other reduced inorganic sulfur compounds (RISCs), resulting in the generation of ferric iron and sulfuric acid. Ferric iron serves as an oxidant of sulfides under acidic conditions, during which the insoluble metal-bearing sulfide minerals are converted into soluble sulfates, releasing the base metals of interest. On contact with atmospheric or dissolved oxygen, copper sulfide ores are oxidized into copper sulfate, sulfuric acid, and iron sulfate (Schippers et al., 2014), a process that is greatly accelerated by the actions of chemolithotrophs (such as *A. ferrooxidans*). All the above compounds (sulfuric acid and copper/ferric sulfates, but also ferrous sulfate) present the major electrolytes in copper bioleaching solutions.

Many researchers found that oxygen solubility in liquid medium was affected by the partial pressure of oxygen, properties of oxygen, composition of the liquid medium, and temperature (Tromans, 2000a; Geng and Duan, 2010; Haibara et al., 2014). Oxygen concentration decreased when ionic solutes presented in solution due to salting-out effect (Cui and Wu, 2013). Most researchers measured dissolved oxygen concentration in seawater or ionic liquids (Weiss, 1970; Song et al., 2019). There is a lack of models to accurately predict the saturated dissolved oxygen concentration in solutions during the bioleaching process of copper sulfide ores.

The Sechenov salt-effect parameter is often considered in theoretical models for gas concentration in electrolyte-containing solutions (Clever, 1983). Sechenov tested the equation by measuring the solubility of carbon dioxide in a concentrated (sometimes saturated) electrolyte solution, a number of dilutions of the solution, and pure water. It revealed the relationship between oxygen solubility and ionic solute in liquid medium. However, this equation can only be used to predict the oxygen solubility in solutions containing one electrolyte. Tromans’ equation is different from Sechenov’s equation in that multiple solutes are considered (Tromans, 2000a). Based on a thermodynamic analysis of water, Tromans proposed a model for estimating the concentration of oxygen in water and inorganic electrolyte solutions. However, the conjoint effect of temperature, pH, and ions concentration of main electrolytes on dissolved oxygen concentration remained indistinct.

Researchers found that the salt-effect parameters calculated from various concentrations differ negligibly, if they differ at all (Clever, 1983). However, oxygen solubility differs in various solutions. Iron ions are particularly important among various metal ions in the growth environment of *A. ferrooxidans* (Kim et al., 2021). Fe^2+^ is a necessary component of growth media from which Fe^3+^-rich leaching liquors are generated. However, excessive Fe^3+^ may result in the formation of a passivation layer comprising ferric precipitates (such as jarosite), thus hindering the bioleaching process (Yu et al., 2011). For this reason, oxygen solubility was measured in test solutions that were characteristic of the copper sulfide ore bioleaching solutions. The variables were designed to highly reduce *in situ* fragmentation bioleaching of copper sulfide ore. Thus, in this research, the metal ions mainly contained in copper sulfide ore bioleaching solutions were Cu^2+^, Fe^2+^, and Fe^3+^.

The objective of this paper is to establish a model for predicting saturated dissolved oxygen concentration in different bioleaching periods of copper sulfide ores. Firstly, based on the growth characteristics of *A. ferrooxidans* and the high temperature characteristics of *in situ* fragmentation bioleaching, the design range of the variables (temperature, pH, and concentrations of ferrous sulfate, ferric sulfate, and copper sulfate) was determined. The growth characteristics of *A. ferrooxidans* were obtained by bacterial domestication and cultivation tests. Secondly, a test device for dissolved oxygen concentration under conditions of simulated forced ventilation was designed and used in experiments. According to its orthogonal experimental design, 25 groups of tests were performed. Then, Python programming language was used to analyze the experimental data using the least squares method to obtain the dissolved oxygen concentration model and the surface fitting. This model was then analyzed by multiple linear regression with Python programming language. Additionally, based on the verification experiments, a model with high-fitting accuracy was proposed to predict the saturated dissolved oxygen concentration by measuring temperature, pH, and the concentrations of Fe^2+^, Fe^3+^, and Cu^2+^ in different bioleaching periods of copper sulfide ores. Considering the oxygen demands of chemical reaction and microbial growth, the prediction model of saturated dissolved oxygen concentration in leaching solutions favors forced aeration technology in *in situ* fragmentation bioleaching.

## Materials and Methods

### Minerals and Reagents

The copper sulfide ores used in the experiments were obtained from Zijinshan copper mine in Fujian, China. *A. ferrooxidans* was domesticated and cultivated in the acidic solution taken from the State Key Laboratory of Comprehensive Utilization of Low-Grade Refractory Gold Ores, Zijin Mining Group Co. Ltd., China. The growth temperature range of the *A. ferrooxidans* strain was 28–45°C. The 9K medium was used for enrichment culture of microorganisms in acidic solutions. It was formed by uniformly mixing liquid A and liquid B. Liquid A contained K_2_HPO_4_ 0.5 g/L, KCl 0.1 g/L, (NH)_2_SO_4_ 3 g/L, MgSO_4_⋅7H_2_O 0.5 g/L, and Ca(NO_3_)_2_ 0.01 g/L; 1:1 H_2_SO_4_ was used to adjust the pH to 2.0. Liquid B contained FeSO_4_⋅7H_2_O 44.3 g/L; 1:1 H_2_SO_4_ was used to adjust the pH to 2.0, and a 0.22-μm Millipore filter was used for sterilization. Liquid A was autoclaved in a vertical pressure steam sterilizer (Shenan Medical Equipment Instrument, China) at 121°C for 20 min (Xiong and Guo, 2011). All reagents were purchased from Aladdin Industrial, China. Pure water, prepared by passing deionized water through a laboratory water purification system, was used in the experiments.

### *Acidithiobacillus ferrooxidans* Domestication and Cultivation Tests

*Acidithiobacillus ferrooxidans* domestication and cultivation tests were performed to obtain their growth characteristics. Firstly, the copper sulfide ore samples were crushed into particles smaller than 5 mm by a Jaw Crusher (Chenggong Mining Equipment Instrument, China). Secondly, the crushed samples (20 g) were taken into a 250-ml conical flask, and 100 ml of 9K medium was added. Then a sterile cotton stopper was used to plug the mouth of the conical flask, and the flask was placed in a constant-temperature incubator with a rotary speed of 90 rev/min at 30°C. Sulfuric acid was added to keep the solution pH at 1.6. Fe^2+^ and Fe^3+^ concentrations were monitored for solution color change. Lastly, domestication tests were concluded when the solution color turned red and the Fe^2+^ oxidation rate was > 80%.

After the domestication tests, 90 ml of sterile 9K medium and 10 ml of the cultured bacteria were placed in a 250-ml conical flask. Sulfuric acid was added to keep the pH at 1.8. The culture solutions were placed in a constant-temperature incubator with a rotary speed of 150 rev/min at 30°C. Culturing solution pH was measured every 24 h (see Supplementary Material 1). A culture color that turned from light blue to yellow brown with precipitates indicated that a majority of the Fe^2+^ present were bio-oxidized to Fe^3+^. Finally, the domestication culture with the darkest color and the highest Fe^2+^ conversion rate was subcultured for nine generations.

After the cultivation tests, the *A. ferrooxidans* (see Supplementary Material 2) used in the experiments was obtained. Its growth cycle was 2 days, the optimal growth pH range was 2–2.4, and the growth temperature range was 28–45°C. Compared with the appearance and growth characteristics of bacteria in the study of Jia et al. (2019), *A. ferrooxidans* dominated the bacterial communities. Thus, growth conditions of *A. ferrooxidans* were considered in designing pH and temperatures in dissolved oxygen concentration measurements.

### Experimental Setup for Dissolved Oxygen Measurements in *Acidithiobacillus ferrooxidans* Cultures

**FIGURE 1:**
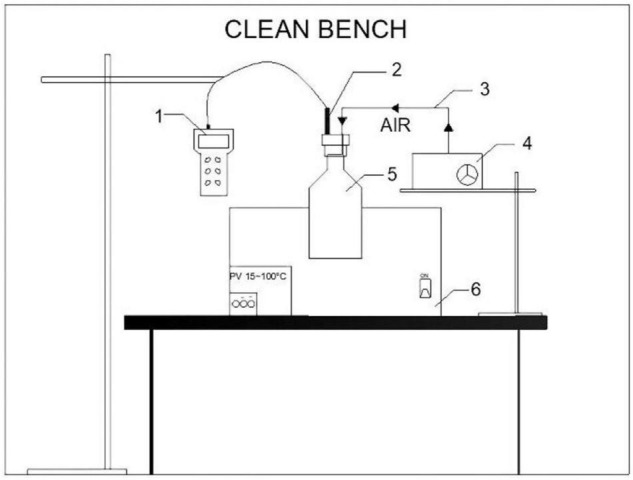
Experimental setup for measuring saturated dissolved oxygen concentration in bioleaching solutions (1—dissolved oxygen meter; 2—dissolved oxygen electrode; 3—silicone hose; 4—oxygen pump; 5—dissolved oxygen measuring flask; 6—thermostat water bath).

The whole setup is shown in Figure 1. Flasks for measuring dissolved oxygen concentration were immersed in a thermostat water bath to guarantee the targeted temperatures. Experiments were conducted under the following conditions:

(1) Temperature. The growth temperature range of *A. ferrooxidans* is 28–45°C, and the growth of *A. ferrooxidans* is inhibited above 50°c. Therefore, the temperature range was set to 30–50°C.
(2) Constant air pressure. To prevent possible gas leakage during oxygen pumping, the oxygen pump and the dissolved oxygen measuring flask were connected by a silicone hose. A hole with the same diameter as the silicone hose was drilled throughout the flask cork.
(3) Oxygen saturation. To ensure oxygen saturation in bioleaching solutions, air was pumped with an oxygen pump for at least 30 min.
(4) Sterile. To prevent the interference of other bacteria and impurities, the measurements were performed in a sterile environment.

### Saturated Dissolved Oxygen Concentration Tests in Abiotic Leaching Solutions

The orthogonal experimental design with five factors and five levels was used in the experiments. Influential factors included temperature, pH, and Fe^2+^, Fe^3+^, and Cu^2+^ concentrations. The saturated dissolved oxygen concentration of bioleaching solutions was measured at combinations of each factor and each level. The iron ions concentration of the bioleaching solutions was controlled according to the following conditions. According to the real bioleaching production parameters, the Fe^3+^ and Cu^2+^ concentration ranges are 0–8 and 0–10 g/L, respectively (Vargas et al., 2020). The headers of the five-factor and five-level orthogonal design are listed in Table 1.

**TABLE 1:** Headers of the five-factor and five-level orthogonal design.

No.	Temperature (°C)	pH	[Fe^2+^] (g/L)	[Cu^2+^] (g/L)	[Fe^3+^] (g/L)
1	30	1.5	0	0	0
2	35	2	1	3	1
3	40	2.5	3	5	3
4	45	3	5	8	5
5	50	3.5	8	10	8

The saturated dissolved oxygen concentration of bioleaching solutions was measured in CuSO_4_–FeSO_4_–Fe_2_(SO_4_)_3_–H_2_SO_4_–H_2_O solutions. Thus, the pH and metal ions concentration ranges of the test solutions were characteristic of the copper sulfide ore bioleaching solutions. The test solutions were prepared by dissolving copper sulfate pentahydrate, ferrous sulfate pentahydrate, and ferric sulfate in pure water. First, to ensure that ferric sulfate was completely dissolved in water and the desired pH was reached, 1:1 sulfuric acid was added to the solutions appropriately. Since ferric sulfate, ferrous sulfate, and copper sulfate were strong acid electrolytes, solution pH was always lower than the designed values. Then, the solutions were filtered through a 0.22-μm Millipore filter. Finally, the air was pumped with an oxygen pump for at least 30 min, and the measuring flask was placed in a thermostat water bath until it reached the desired temperatures. The whole setup was placed inside a clean bench under a constant-temperature condition.

### Column Bioleaching Verification

The model designed for oxygen concentration in copper bioleaching solutions was verified by a column bioleaching test. The leaching column was an acrylic glass column with a double-ring structure. The temperature of the leaching system was maintained at 30–50°C by circulating water at a controlled temperature between the inner and the outer walls of the column. The column vertical profile comprised three parts: (i) the top 3 cm of the column was filled with cobblestones 5 mm in diameter, serving as a filter layer; (ii) the middle 50 cm of the column was filled with ore particles < 8 mm in diameter; and (iii) the bottom 10 cm of the column was a gas-stable chamber. The column was sealed, except for the inlet and outlet solution pipes, to prevent atmospheric air disturbance. The leisurely spraying systems were conducted, i.e., 3-day spraying and 1-day suspending in the initial stage, and 2-day spraying and 2-day suspending in the later stage. The bacterial leaching solution was added to the column at a rate of 4 ml/min. The column bioleaching test was carried out for 36 days.

### Analytical Methods

Dissolved oxygen concentration was determined using the HengXin AZ-8403 dissolved oxygen sensor (AZ Instrument, China) connected to a meter with temperature and pressure compensation. The temperature range of the dissolved oxygen meter was 0–50°C, which meets the temperature requirements. This device also fulfills continuous measurement of dissolved oxygen concentration at a resolution of 0.01 mg/L and a relative accuracy of 1.5%. The thermostat water bath (Xinbao Instrument, China) was used to control the temperature between 30 and 50°C. Oxygen was pumped through an oxygen pump (Songbao Instrument, China), which can adjust the air output until the solution reaches oxygen saturation (transition time was approximately 30 min). Solution pH was measured with the STARTER 2100 pH meter (Ohaus Instrument, United States) equipped with an ST310 electrode. Panreac buffer solutions at pH 4, 6.86, and 9.18 were used for calibration. The metal ions concentrations in the solutions were measured by an inductively coupled plasma emission spectrometer. All measurements were performed on a clean bench with a clean class of ISO 5 (Class 100).

## Results and Discussion

### Establishment and Analysis of Dissolved Oxygen Concentration Model

Dissolved oxygen concentration is commonly measured at 30°C and 1 atm P_O_2__ to explore the effects of inorganic solutes on oxygen solubility in various solutions (Tromans, 2000b). In order to explore the conjoint effects of inorganic ions concentration, temperature, and pH on oxygen solubility, the measurements were performed within temperature and pH ranges that were similar to *in situ* copper sulfide ore bioleaching. Python language is commonly used in data processing and analysis. Through data training, complex algorithms such as data fitting, regression prediction, clustering, and model selection can be easily realized. Especially in surface fitting, Python language has high flexibility and efficiency. Thus, based on the least squares method, Python programming language was used to analyze 25 sets of measurement data in orthogonal experiments (Table 2), and the saturated dissolved oxygen concentration model was obtained as shown in Eq. 1:


(1) $$Y=9.72-8.31\times 10^{-2}\,X_{1}-3.71=10^{-4}\,X_{2}-7.06=10^{-3}\,X_{3}-2.47\times 10^{-2}\,X_{4}-9.75\times 10^{-3}\,X_{5}$$

**TABLE 2:** Saturated dissolved oxygen concentration measurements.

No.	Temperature (°C)	pH	[Fe^2+^] (g/L)	[Cu^2+^] (g/L)	[Fe^3+^] (g/L)	Oxygen concentration (mg/L)
						
1	30	1.5	0	0	0	7.14
2	30	2	1	3	1	7.2
3	30	2.25	3	5	3	7.05
4	30	2.27	5	8	5	6.82
5	30	2.41	8	10	8	6.51
6	35	1.5	1	5	5	6.73
7	35	2	3	8	8	6.69
8	35	2.5	5	10	0	6.46
9	35	3	8	0	1	6.75
10	35	2.61	0	3	3	6.70
11	40	1.5	3	10	1	6.55
12	40	2	5	0	3	6.30
13	40	2.5	8	3	5	6.21
14	40	2.28	0	5	8	6.18
15	40	3.23	1	8	0	5.97
16	45	1.5	5	3	8	5.95
17	45	2	8	5	0	5.90
18	45	2.5	0	8	1	5.50
19	45	2.44	1	10	3	5.72
20	45	2.26	3	0	5	5.60
21	50	1.5	8	8	3	5.62
22	50	2	0	10	5	5.29
23	50	2.17	1	0	8	5.57
24	50	3	3	3	0	5.30
25	50	2.82	5	5	1	5.52

where *Y* is saturated dissolved oxygen concentration (mg/L), *X*_1_ is temperature (°C), *X*_2_ is pH, *X*_3_ is Fe^2+^ concentration (g/L), *X*_4_ is Cu^2+^ concentration (g/L), and *X*_5_ is Fe^3+^ concentration (g/L).

**FIGURE 2:**
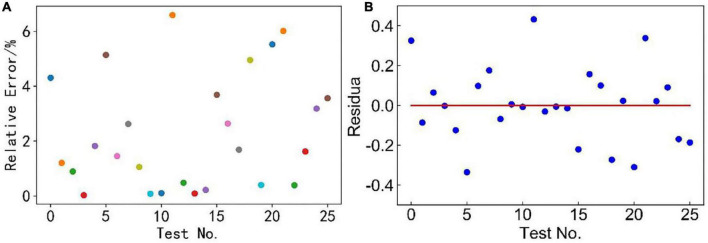
**(A)** Relative error and **(B)** residual value of the dissolved oxygen concentration model.

Based on the theory of multi-linear regression analysis, the fitting parameters analyses of this model were performed by Python programming language, as shown in Figure 2 and Table 3. Results show that the relative error of 76% measurements was < 4%, mostly around 1% (Figure 2A), and the residuals were randomly distributed around the zero scale line (red line in Figure 2B), with most of them between –0.2 and 0.2 without abnormal points. The fitting parameters show that this model had a high-fitting accuracy, as shown in Table 3. *P* < 0.05 indicated that the regression coefficient was significant. *F* > *F*_0.95_(5, 20) = 4.56 indicated that the regression equation was significant (Huang and Wu, 2020).

**TABLE 3:** Fitting parameters of this model.

Fitting parameters	Sum squared residual	Average relative error	F	P	R^2^
Value	0.034	0.0226	42.83	< 0.0001	0.966

**FIGURE 3:**
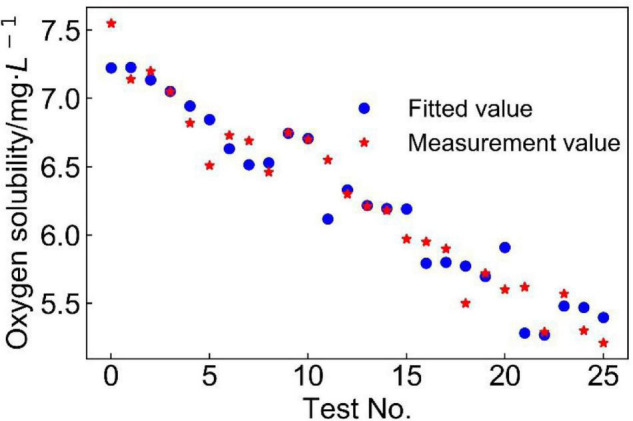
Comparison of experimental (in red) and fitted (in blue) values of dissolved oxygen concentration in copper bioleaching solutions.

To test the goodness-of-fit of the model, the designed value of each group was substituted into Eq. 1, and comparison between designed values and measured values shows that this model had a high-fitting accuracy (Figure 3). It shows that a majority of the predicted values fitted very closely to the experimental dissolved oxygen concentrations, with the five factors (temperature, pH, and Fe^2+^, Cu^2+^, and Fe^3+^ concentrations; values summarized in Table 2) showing a significant linear correlation with oxygen concentration. This effect shows that dissolved oxygen concentration decreased with increasing temperature and ion concentration, which was characteristic of salting-out effect. At 30°C–40°C (test nos. 1–15), the model had a higher prediction of dissolved oxygen concentration. The temperature growth range of mesophilic leaching bacteria (such as *A. ferrooxidans*) is generally accepted to be between 30 and 40°C (Niemel et al., 1994), which correlates well with the characteristics of the developed model.

Compared with previous studies (Clever, 1983; Tromans, 2000b; Mazuelos et al., 2017), this model had a higher accuracy in predicting the saturated dissolved oxygen concentration of bioleaching solutions in different leaching periods of copper sulfide ore bioleaching. To analyze the independent effect and the conjoint effects of the five factors on oxygen solubility, Python programming language was used for further analysis.

### Comparison of Correlation Coefficients of Independent Factors

A thorough understanding of the effects of temperature, as well as other factors, on dissolved oxygen concentration enables the leaching processes to be modeled and controlled more effectively (Tromans, 1998). Temperature is generally considered to have a great effect on oxygen solubility (Mehdizadeh and Ashraf, 1900; Geng and Duan, 2010; Fleige et al., 2016). Tromans proposed a correction of *T* by measuring the oxygen solubility in water when the reference temperature was 30°C (Mazuelos et al., 2017), as shown in Eq. 2:


(2) $${\text{S}}_{\text{T}}^{0}={\text{S}}^{0}\cdot {\text{e}}^{\left(\frac{1336}{273.15+\text{T}}-\frac{1336}{303.15}\right)}$$

where *T* is the temperature in degrees Celsius (°C) and *S*_T_^0^ is the reference solubility measured at a temperature other than 30°C, which differs greatly from *S*^0^ (oxygen solubility in pure water at 30°C and 1 P_O_2__ atm, generally considered as 7.56 mg/L).

**FIGURE 4:**
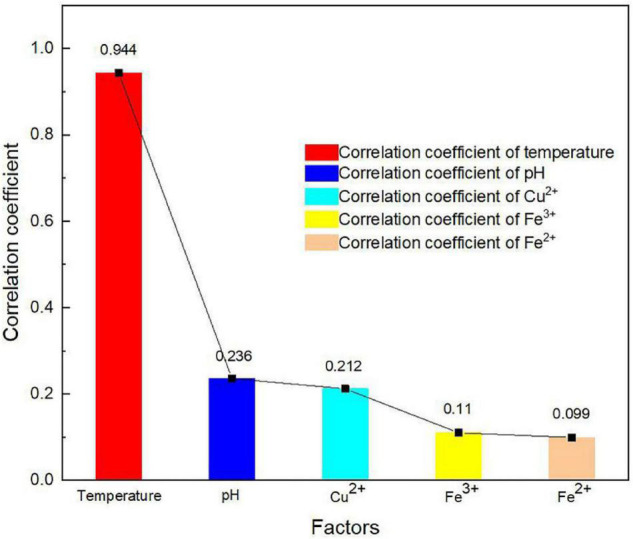
Correlation coefficient of factors to dissolved oxygen concentration.

The correlation coefficient of temperature in an oxygen solubility model is an important parameter to consider. For this reason, the correlation coefficient of the independent factors to dissolved oxygen concentration was obtained by performing linear regression analysis, as shown in Figure 4. The algorithm of the correlation coefficient is as follows:


(3) $$r\,\left(X,Y\right)=\frac{\text{Cov}\,\left(\text{X},\text{Y}\right)}{\sqrt{\text{Var}\,\left|\text{X}\right|\,\text{Var}\,\left|\text{Y}\right|}}$$

where *r*(*X*,*Y*) is the correlation coefficient of a factor to the dissolved oxygen concentration; Cov(*X*,*Y*) is the covariance between the designed value and the measurement value; Var| *X*| and Var| *Y*| are the variance of the designed value and the measurement value, respectively, which can be obtained by substituting the designed value into the Python algorithm.

The correlation coefficient of temperature to dissolved oxygen concentration was significantly greater than those of other parameters, reaching the value of 0.944 (Figure 4). It indicates that temperature had a more significant effect on oxygen concentration than other factors. As many researchers considered, the effect of pH on oxygen solubility is not significant (Mazuelos et al., 2017). It is worth pointing out that dissolved oxygen concentration decreased linearly as proton concentration in the solutions increased. Figure 4 shows that the changes in Fe^2+^ and Fe^3+^ concentrations had lesser effects on the dissolved oxygen concentration in bioleaching solutions than that of Cu^2+^. It is considered that the link between bioleaching and ferrous ion bio-oxidation restricted the changes in Fe^2+^ concentration, Fe^3+^ concentration, and pH values (Mazuelos et al., 2017). The bioleaching behavior of chalcocite was used as an example to explore this effect by means of bio-oxidation stoichiometry, as shown in Eqs. 4 and 5:


(4) $$4\,F\,{\text{e}}^{2+}+{\text{O}}_{2}+4\,H^{+}\overset{\text{bacteria}}{⟶}4\,F\,{\text{e}}^{3+}\,2\,{\text{H}}_{2}\,\text{O}$$

(5) $${\text{Cu}}_{2}\,\text{S}+4\,F\,{\text{e}}^{3+}\to 2\,{\text{Cu}}^{2+}+4\,{\text{Fe}}^{2+}\,\text{S}$$

It is generally accepted that chalcocite is easy to oxidize and dissolve compared to other copper sulfides. Additionally, ferrous ions dissolve preferentially to copper ions (Huang, 2015). In *in situ* fragmentation bioleaching at pH 2–3, Fe^3+^ exists predominantly in the form of precipitates. As mentioned in the Introduction, Fe^2+^ and Fe^3+^ are in a continuous oxidation–reduction process and copper is continuously leached through the action of bacteria. This is the result of a dynamic balance between the bacterial growth and the change in iron ions concentration (Huang, 2015). There are two mechanisms for sulfide ore bioleaching: one is that microorganisms are directly adsorbed on the surface of ore particles and promote mineral oxidation through biological enzymes; the other is that microorganisms directly oxidize Fe^2+^ to Fe^3+^, so as to provide oxidants for mineral oxidation reaction. The dissolution of minerals is completed through the thiosulfate pathway or polysulfide compound pathway (Romano et al., 2001). Bioleaching of sulfide ore is generally a chemical reaction process combining the attack by protons and Fe^3+^. RISCs and Fe^2+^ are used as electron donors by microorganisms in the bioleaching process. Fe^3+^ and oxygen are used as electron acceptors (Rawlings, 2005). For pyrite bioleaching, when Fe^2+^ is oxidized by bacteria, the cytochrome in the bacteria is used as the electron transfer chain, and the molecular oxygen or oxygen-containing layer on the mineral surface is used as the final electron acceptor.

### Comparison of Multi-Correlation Coefficients of Various Factor Combinations

To explore the effects of various factor combinations on dissolved oxygen concentration, the multi-correlation coefficient of various factor combinations was calculated by linear regression analysis. The algorithm of the correlation coefficient is as follows:


(6) $$\text{R}=\text{corr}\left(Y,X_{1},,,X_{5}\right)=\text{corr}\left(Y,\hat{Y}\right)=\frac{\text{Cov}\,\left(Y,\hat{Y}\right)}{\sqrt{\text{Var}\,\left|Y\right|\,\text{Var}\,\left|\hat{Y}\right|}}$$

where $\text{corr}\left(Y,\hat{\text{Y}}\right)$ is an automatic calculation function in Python programming language; $\hat{\text{Y}}$ is the equation obtained by linear regression of the measurement value to the designed value; $\text{Cov}\left(Y,\hat{\text{Y}}\right)$ is the covariance between the measurement value and the fitted value; and Var| *Y*| and $\text{Var}\left|\hat{\text{Y}}\right|$ are the variance of the measurement value and the fitted value, respectively, which can be obtained by performing the Python algorithm.

**FIGURE 5:**
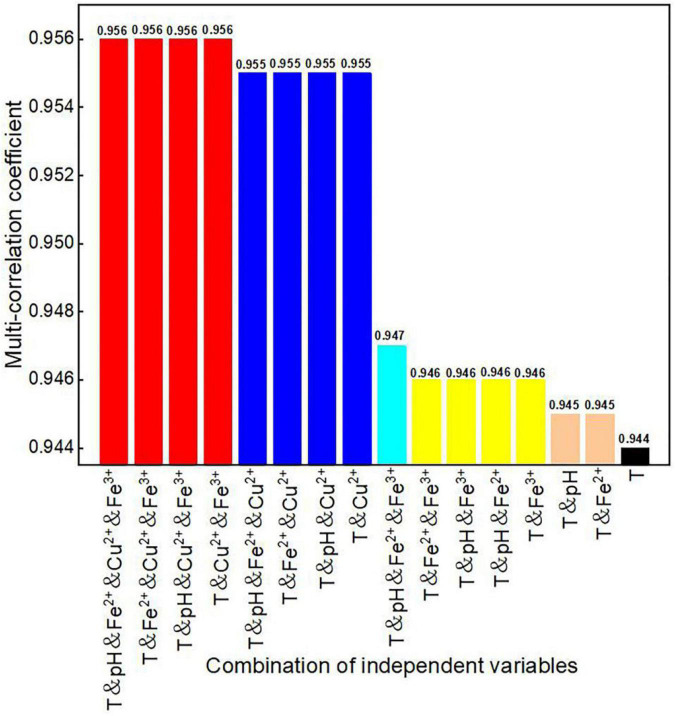
Multi-correlation coefficients of various combinations of process parameters.

**FIGURE 6:**
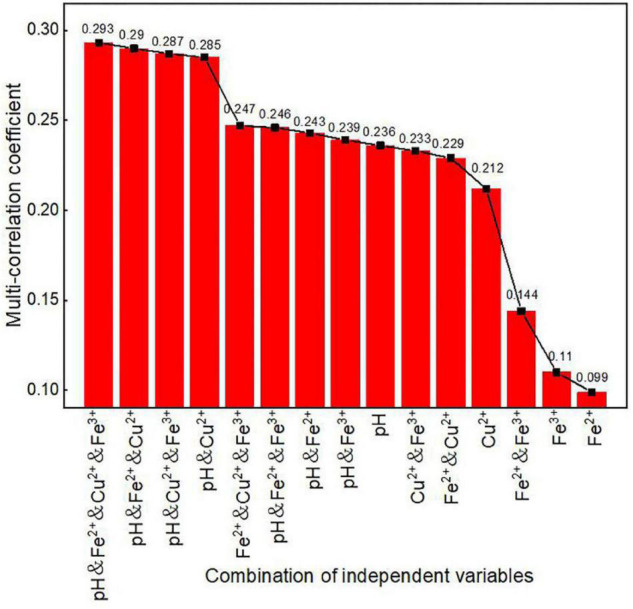
Multi-correlation coefficients of factor combinations without temperature.

In the analysis, the multi-correlation coefficients of various factor combinations (Figure 5) and the multi-correlation coefficients of the factor combinations without temperature (Figure 6) were calculated separately. The reason for separate analyses with and without temperature was to directly explore the degree of influence that temperature has on dissolved oxygen concentration in *in situ* fragmentation bioleaching systems.

When considering temperature, the multi-correlation coefficients of various factor combinations to dissolved oxygen concentration were > 0.944. It indicates that temperature was the most significant factor affecting the dissolved oxygen concentration (Figure 5). Figure 5 shows that the multiple correlation coefficients without Cu^2+^ were significantly reduced (e.g., from the maximum value of 0.956–0.947, for the same factor combination but without Cu^2+^). This indicates that Cu^2+^ concentration had the greatest influence among the three ion concentrations considered in this model.

The multi-correlation coefficients were significantly lower when temperature was not considered (Figure 6), compared to those with temperature. There were two prominent drops in the graph when pH and Cu^2+^ concentrations were not considered. However, when both pH and Cu^2+^ concentrations were considered, the maximum multi-correlation coefficients varied between 0.285 and 0.293, indicating that these two factors had lesser effects on oxygen concentration than temperature.

### Conjoint Effects of Temperature and Other Factors on Dissolved Oxygen Concentration

In the process of bioleaching, temperature had a significant effect on bacterial growth and dissolved oxygen concentration. According to the previous analysis, the change in temperature and other four factors affected the dissolved oxygen concentration in bioleaching solutions. To explore the conjoint effects of the factors on dissolved oxygen concentration, temperature, as the main factor, was combined with other factors to obtain surface fitting based on the least squares method. The algorithm of the least squares method is as follows:


(7) $$\min f\,\left(X\right)=\sum_{i=1}^{m}{\left[Y_{i}-f\,\left(X_{i},w_{i}\right)\right]}^{2}$$

where *m* is the sample size of the experiments (value is 25); *X*_i_ and *Y*_i_ are the designed value and measurement value of each group, respectively; and *w*_i_ is the parameter that needs to be determined to minimize the value of the above function.

**FIGURE 7:**
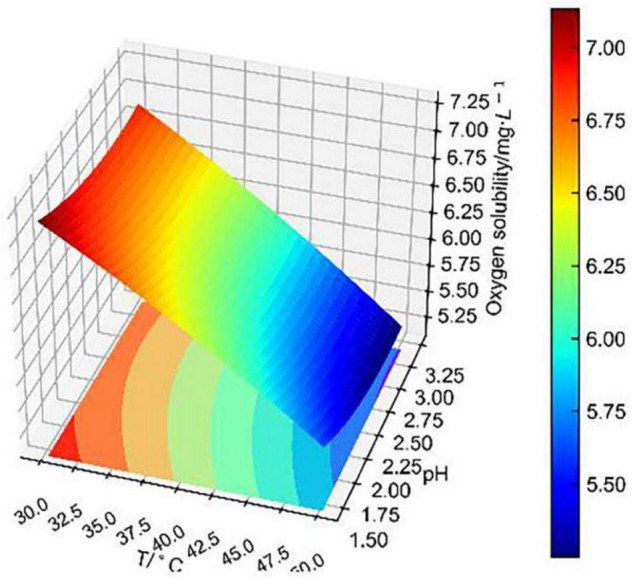
Conjoint effect of temperature and pH on dissolved oxygen concentration.

**FIGURE 8:**
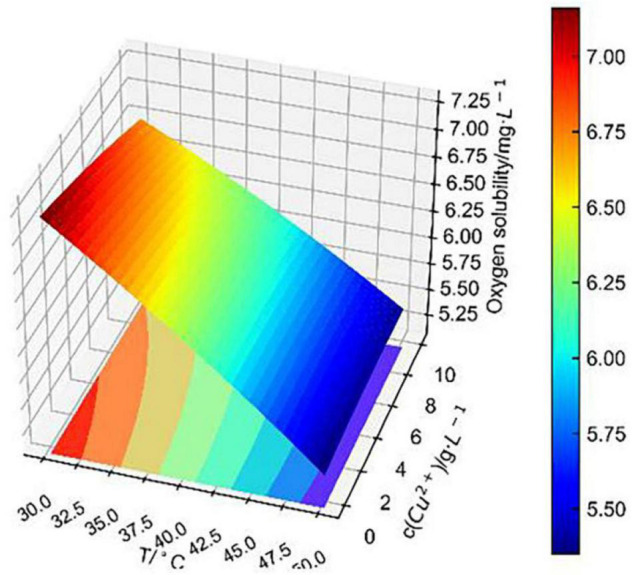
Conjoint effect of temperature and Cu^2+^ concentration on dissolved oxygen concentration.

**FIGURE 9:**
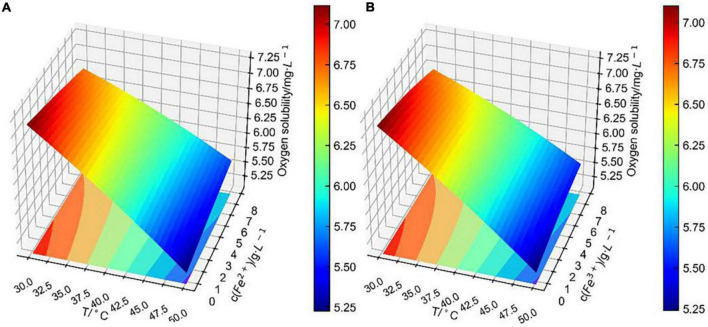
Conjoint effect of **(A)** temperature and **(B)** iron ions concentration on dissolved oxygen concentration.

Based on the theory of the L–S method, the binary surface approximation model was loaded in the Python database, and the experimental data were substituted into the binary surface parameter matrix. Finally, the fitting surface of two combined factors to dissolved oxygen concentration was obtained as shown in Figures 7–9.

Figure 7 shows that the fitting surface of temperature and pH to dissolved oxygen concentration was steeply inclined. It indicates that dissolved oxygen concentration decreased significantly with the increase in temperature. At 30–40°C, dissolved oxygen concentration decreased with the increase in pH. This effect was different from Alfonso‘s equation, which considered that dissolved oxygen concentration decreased with the decrease in pH (Mazuelos et al., 2017). That is because Alfonso measured dissolved oxygen concentration in an aqueous solution of sulfuric acid, which only considered the change in pH. However, for this paper, dissolved oxygen concentration was measured in CuSO_4_–FeSO_4_–Fe_2_(SO_4_)_3_–H_2_SO_4_ solutions at 30–50°C. Especially considering the significant effect of bioleaching temperature, the effect of pH on dissolved oxygen concentration is hard to predict. It is certain that pH is linearly related to dissolved oxygen concentration, which is consistent with other studies (Mazuelos et al., 2017; Li et al., 2021). Although pH had little effect on dissolved oxygen concentration, the conjoint effect with temperature was significant, which was consistent with the previous independent effect analysis.

Figure 8 shows that the fitting surface of temperature and Cu^2+^ concentration to dissolved oxygen concentration was smoother than that of temperature and pH, indicating that the change in pH had a greater effect on dissolved oxygen concentration than that of Cu^2+^ concentration, which was consistent with the previous analysis of independent effect. It is obvious that the change in Cu^2+^ concentration had less effect on dissolved oxygen concentration as temperature exceeded 40°C. At 30–40°C, the change in Cu^2+^ concentration at each temperature level had a significant linearly positively correlated effect on dissolved oxygen concentration.

It is obvious that the conjoint effects of the fitting surface of Fe^2+^ concentration with temperature (Figure 9A) and that of Fe^3+^ concentration with temperature (Figure 9B) differed negligibly. As previously analyzed, the correlation coefficient of Fe^2+^ concentration to dissolved oxygen concentration was similar to that of the Fe^3+^ concentration. It is worth pointing out that the change in iron ions concentration had less effect on dissolved oxygen concentration than that of Cu^2+^ concentration within the temperature range from 30 to 50°C, which was concluded by comparing Figures 8, 9. However, in the direct or indirect effects of bacteria on bioleaching, iron ions are involved in an important role (Sasaki et al., 2011). The Fe concentration in bioleaching solutions increased with the increase of bioleaching cycle. Therefore, in each bioleaching cycle, the changes in temperature, pH, and ions concentration in bioleaching solutions need to be carefully considered, and the model (Eq. 1) can be used to accurately predict the dissolved oxygen concentration in bioleaching solutions.

### Model Verification Test

In order to verify the abovementioned model (Eq. 1), the pregnant leaching solutions were taken from the column bioleaching test on days 4, 8, 12, 16, 20, 24, 28, 32, and 36. Table 4 shows that the experimental measurements were very close to the predicted values, with the maximum absolute error in oxygen concentration reaching 0.09 mg/L. This indicates that the proposed model (Eq. 1) shows great accuracy in predicting dissolved oxygen concentration and saturation in bioleaching solutions in different time periods during *in situ* fragmentation bioleaching of copper sulfide ore.

**TABLE 4:** Results of verification tests (*C*_I_ is the measurement value and *C*_Eq. 1_ is the value predicted by Eq. 1).

Day	Temperature (°C)	pH	Cu^2+^ (g/L)	Fe^2+^ (g/L)	Fe^3+^ (g/L)	*C*_I_ (mg/L)	*C*_Eq. 1_ (mg/L)
4	30	2.18	0.162	3.284	0.365	7.18	7.2
8	30	2.05	0.339	5.755	0.639	7.21	7.17
12	35	1.97	0.541	6.871	1.718	6.81	6.73
16	35	1.92	0.853	7.895	3.383	6.67	6.7
20	40	1.89	0.934	8.401	4.524	6.24	6.27
24	40	1.74	1.149	8.6286	5.752	6.29	6.25
28	45	1.69	1.258	7.549	7.549	5.78	5.82
32	45	1.62	1.322	6.824	9.424	5.72	5.81
36	50	1.61	1.335	5.412	12.047	5.47	5.38

## Conclusion

Among different process parameters (temperature, pH, and concentrations of ferrous sulfate, ferric sulfate, and copper sulfate), temperature had the most significant effect on dissolved oxygen concentration. The dissolved oxygen concentration decreased linearly with the increase in temperature. Variations in Cu^2+^ concentration had a more significant effect on the dissolved oxygen concentration than those in Fe^2+^ and Fe^3+^ concentrations. At 30–40°C, dissolved oxygen concentration decreased with increasing metal ions concentrations. When considering temperature, the link between the conjoint effect of the five factors on dissolved oxygen concentration prevented the evaluation of the sole effect of pH, resulting in pH that was linearly negatively related to the dissolved oxygen concentration. Changes in pH had little effect on the saturated dissolved oxygen concentration. A mathematical model of dissolved oxygen concentration was proposed based on surface fitting by Python language, and this model was experimentally verified. This model showed high accuracy in fitting dissolved oxygen concentrations during different periods of copper sulfide ore bioleaching, indicating that in *in situ* fragmentation bioleaching, dissolved oxygen concentration and saturation can be predicted by monitoring the temperature, pH, and Fe^2+^, Fe^3+^, and Cu^2+^ concentrations in the bioleaching solution.

## Data Availability Statement

The original contributions presented in the study are included in the article/Supplementary Material, further inquiries can be directed to the corresponding author/s.

## Author Contributions

M-QH conceived the study and wrote the first draft of the manuscript. MZ and LC collected the mineral samples. M-QH and Z-LX collected and cultured the microorganisms. M-QH and MZ carried out the saturated dissolved oxygen concentration measurements. MZ, LC, and Z-LX revised the manuscript. All authors read and approved the submitted version.

## Conflict of Interest

The authors declare that the research was conducted in the absence of any commercial or financial relationships that could be construed as a potential conflict of interest.

## Publisher’s Note

All claims expressed in this article are solely those of the authors and do not necessarily represent those of their affiliated organizations, or those of the publisher, the editors and the reviewers. Any product that may be evaluated in this article, or claim that may be made by its manufacturer, is not guaranteed or endorsed by the publisher.
