# Supplementary material for: Saturated Dissolved Oxygen Concentration in in situ Fragmentation Bioleaching of Copper Sulfide Ores
Source: Front Microbiol. 2022 Apr 6;13:821635. doi: 10.3389/fmicb.2022.821635 (PMC9019730; doi:10.3389/fmicb.2022.821635)

**Supplementary Material 1** **|** The pH changes in liquid *At. ferrooxidans* cultures

**
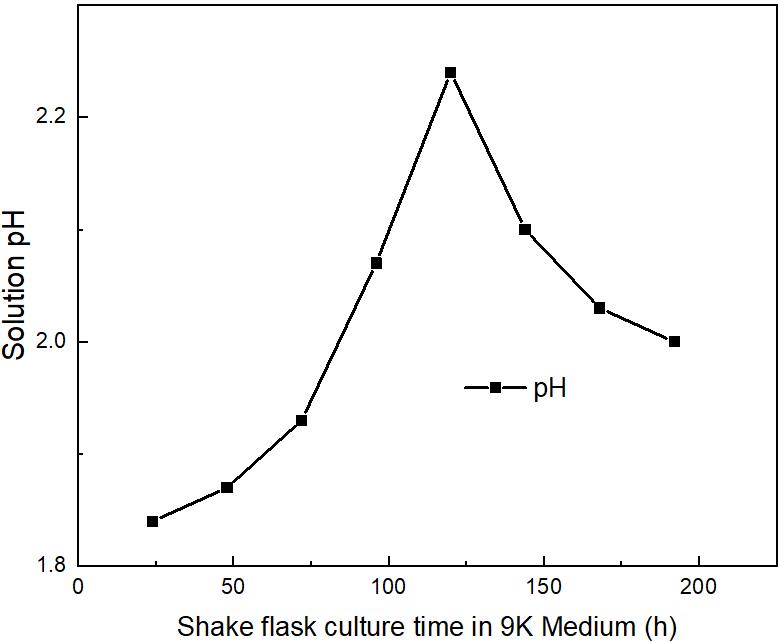
**

**Supplementary Material 2 |** Growth appearence of *At. ferrooxidans* domesticated and cultured from State Key Laboratory of Comprehensive Utilization of Low-Grade Refractory Gold Ores, China.


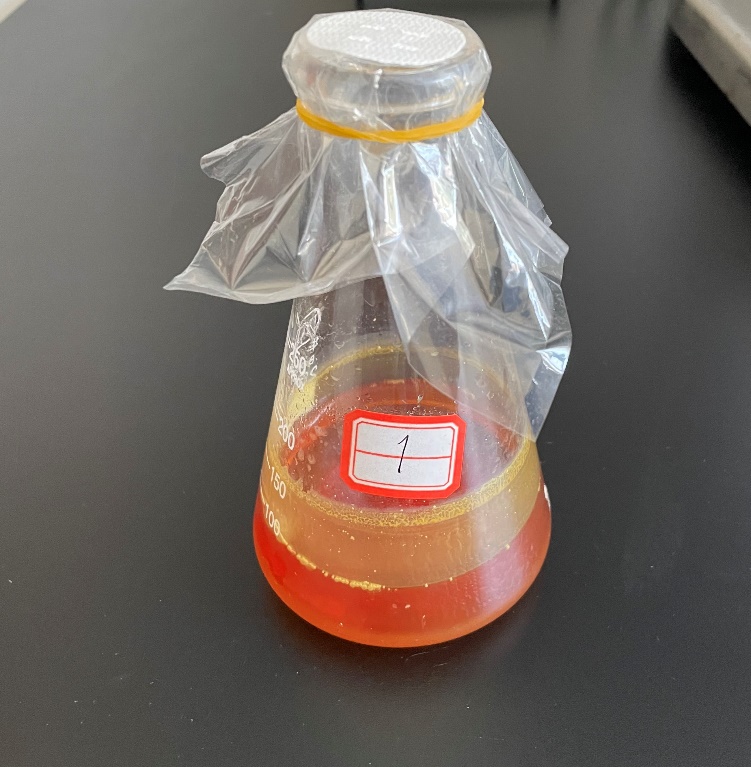

Supplement: Supplementary file 1 [file Data_Sheet_1.docx]
